# Hepatoprotective potential of *Decalepis hamiltonii* (Wight and Arn) against carbon tetrachloride-induced hepatic damage in rats

**DOI:** 10.4103/0975-7406.72137

**Published:** 2010

**Authors:** R. Harish, T. Shivanandappa

**Affiliations:** Department of Microbiology, Pooja Bhagavat Memorial Mahajana Post Graduate Centre, wing of SBRR Mahajana Education Centre, Mysore, India; 1Department of Food Protectants and Infestation Control, Central Food Technological Research Centre, Mysore, Karnataka, India

**Keywords:** Antioxidant activity, CCl_4_, *Decalepis hamiltonii*, hepatoprotective

## Abstract

Hepatoprotective activity of the roots of *Decalepis hamiltonii* (Wight and Arn) was studied using carbon tetrachloride (CCl_4_) induced liver injury model in albino rats. The hepatotoxicity produced by acute CCl_4_ administration was found to be inhibited by pretreating the rats with crude methanolic extract of the roots of *D. hamiltonii* (Dh) prior to CCl_4_ induction. Hepatotoxic inhibition was measured with the decreased levels of hepatic serum marker enzymes (glutamate-pyruvate transaminase (GPT), glutamate oxaloacetate transaminase (GOT), alkaline phosphatase (ALP), and lactate dehydrogenase (LDH) and lipid peroxide formation. Imbalance level of glutathione (GSH) and antioxidant enzymes such as catalase, glutathione peroxidase, and glutathione reductase were normalized in rats pretreated with Dh extract followed by CCl_4_ administration. Pathological changes of hepatic lesions caused by CCl_4_ were also improved by pretreatment with the Dh root extract. The results of this study indicate that roots of *D. hamiltonii* could afford a significant protective action in the alleviation of CCl_4_-induced hepatic damage in rats.

*Decalepis hamiltonii* (Wight and Arn), commonly known as Makali ber in Kannada, belongs to the family Asclepediaceae. It grows largely in southern parts of India in the hilly and forest areas of the Western Ghats. Earlier work on this root has shown to contain aldehydes, alcohols, ketones, sterols, and triterpenes such as amyrin and lupeols derivatives.[1–3] The roots are also used as a substitute for the scarce *Hemidesmus indicus* in the traditional Indian system of medicine because of the similar aromatic properties.[4] Due to the presence of aroma, the roots are consumed as pickles and juices.[5] The dried roots could be stored for long periods without microbial or insect infestation.[6] The antimicrobial properties of the roots of *D. hamiltonii* have been attributed to the presence of 2-hydroxy 4-methoxy benzaldehyde and vanillin.[7] We have recently shown that the roots of *D. hamiltonii* possess antioxidant properties and several bioactive compounds have been isolated and characterized.[8,9] Indian medicinal plants having hepatoprotective potential have been reported in various plants, leaves, and roots.[10–12] Scientific studies of root extract of *D. hamiltonii* as hepatoprotective were lacking; therefore, in this investigation the root extract was tested against CCl_4_ -induced liver injuries to validate its use against hepatic cellular damage.

## Materials and Methods

### Materials

2-Oxoglutaric acid, sodium pyruvate, 2,4-dinitrophenylhydrazine, DL-alanine, L-aspartic acid, *þ*-nitrophenyl phosphate, lactate, nicotinamide adenine dinucleotide (NAD), bovine serum albumin (BSA), trichloroacetic acid (TCA), and hydrogen peroxide (H_2_ O_2_) were procured from Sisco Research Laboratories, India. 1-Chloro-2, 4-dinitrobenzene (CDNB), glutathione (GSH), oxidized glutathione (GSSG), glutathione reductase (GR), cumene hydroperoxide (CHP), thiobarbituric acid (TBA), was purchased from Sigma chemical Co, USA. All other chemicals were purchased from Ranbaxy and Qualigens, India.

### Preparation of root extract

*D. hamiltonii* was procured from the local suppliers in Mysore, India. The taxonomic identification was confirmed from the Department of Botany, University of Mysore. The outer fleshy portion of the roots was separated from the inner hard pith and was cut into small pieces and allowed to dry at room temperature. Dried root material was powdered using grinder and stored in refrigerator till further use. Coarsely powdered dried root material (500 g) was extracted with methanol, using soxhlet apparatus. The methanolic extract obtained was evaporated under reduced pressure to get the dried crude extract.

Animals: Male adult Wistar rats (180–200 g) bred in the animal house of the Institute were caged in uniform hygienic conditions and kept on standard pellet diet and water *ad lib*.

### Single dose pretreatment

Rats were divided into six groups of four animals each. Group 1 (control) was administered orally with the vehicle sunflower oil only (1 ml/kg body weight). Group 2 was administered with the methanolic extract of *D. hamiltonii* alone (200 mg/kg body weight). Group 3 was administered a single dose of CCl_4_ (dissolved in sunflower oil) at 1 ml/kg body weight. Groups 4-6 were pretreated with methanolic extracts of *D. hamiltonii* (50, 100, and 200 mg/kg body weight) followed by the administration of CCl_4_ 1 ml/kg body weight. Rats were sacrificed by ether anesthesia, 24 hr after the treatment.

### Multiple dose pretreatment

Rats were divided into five groups of four animals. Group 1 (control) was administered orally with the vehicle sunflower oil only (1 ml/kg body weight). Group 2 was administered with the methanolic extract of *D. hamiltonii* alone (100 mg/kg body weight) for 7 days. Group 3 served as toxin control (CCl_4_ treated), it was administered with vehicle for 6 days, and on seventh day administered a single dose of CCl_4_ (dissolved in sunflower oil) at 1 ml/kg body weight. Groups 4 and 5 were given with methanolic extracts of *D. hamiltonii* (50, 100 mg/kg body weight) for six consecutive days and with extract + CCl_4_ on seventh day, respectively. All administrations of doses were made orally. Rats were sacrificed by ether anesthesia, after the treatment.

### Serum enzyme assay

Blood was collected by cardiac puncture, allowed to clot and centrifuged at 1000 × *g* to obtain the serum. The enzymes, glutamate-pyruvate transaminase (GPT) and glutamate oxaloacetate tranaminase (GOT), alkaline phosphatase (ALP), and lactate dehydrogenase (LDH) were assayed as described by Bergmeyer.[13] The enzyme activity was expressed as units/liter computed directly from the absorbance values.

### Antioxidant enzymes

Liver homogenate (10% w/v), prepared in 0.1 M phosphate buffer (pH 7.2) was used to assay the enzyme activities. Catalase (CAT) activity was determined according to the method,[14] reduction of H_2_ O_2_ in 0.1 M phosphate buffer (pH 7.2) was estimated kinetically at 240 nm. The activity was calculated using molar absorption coefficient. One unit was defined as amount of the enzyme, which converts 1 mol substrate to product in 1 sec.

Glutathione peroxidase (GPx) activity was determined by the indirect assay method using glutathione reductase. Cumene hydroperoxide (1 M), and glutathione (0.25 mM) were used as substrates and oxidation of NADPH by glutathione reductase (0.25 U) in tris buffer was monitored at 340 nm.[15]

Glutathione reductase (GR) was assayed using oxidized glutathione (2 mM) and NADPH (2 mM) in potassium phosphate buffer.[16]

Glutathione transferase (GST) activity was monitored by the method described by Warholm.[17] using glutathione (2 mM) and CDNB (3 mM) as substrates in phosphate buffer, change in absorbance at 344 nm was monitored.

### Glutathione

GSH content in the liver homogenate (10%) was analyzed according to the method of Ellman[18] Standard GSH was used to calculate the glutathione content, which was expressed in *μ*mol/g liver.

Protein content was estimated by the method of Lowry[19] with BSA as the standard.

### Lipid peroxidation

The liver was removed, washed with 0.9% saline and 10% w/v homogenate was prepared in cold 0.1 M phosphate buffer (pH 7.2). Total lipid peroxide content in the homogenate was assayed by the TBA method.[20] Briefly, to 1 ml of the homogenate was added 1 ml each of 20% TCA and 0.67% TBA solution, mixed thoroughly and heated for 15 min in a boiling water bath. After cooling, centrifuged at 4°C at 2000 rpm for 10 min and the absorbance of the supernatant were read at 535 nm in a spectrophotometer.

### Liver histopathological studies

Liver section taken immediately from the liver, fixed in 10% buffered formalin, dehydrated in ethanol (50–100 %), cleared in xylene, and embedded in paraffin. Sections (4–5 *μ*m thick) were prepared and then stained with hematotoxylin and eosin (H-E) dye for photomicroscopic observation.

#### Statistical analysis

Results were analyzed by Duncan’s multiple range tests, to detect intergroup differences where *P* values <0.05 were considered statistically significant.

## Results

### Antihepatotoxic activity

**Figure 1:**
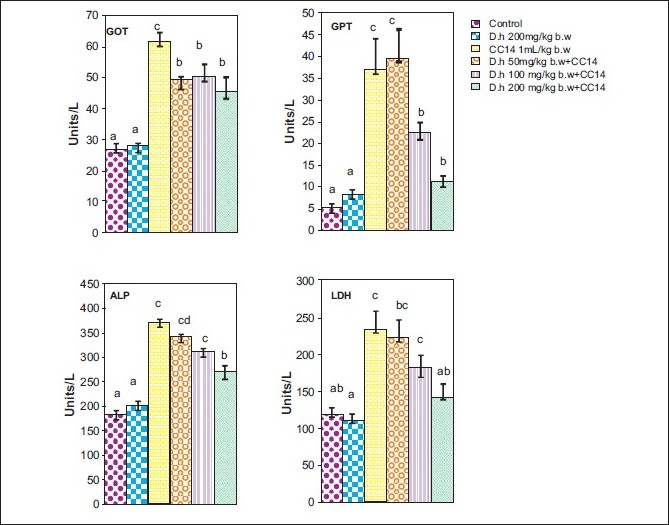
Hepatoprotective action of methanolic extract (single dose) of *D. hamiltonii* against CCl_4_ hepatotoxicity: serum enzyme profile

**Figure 2:**
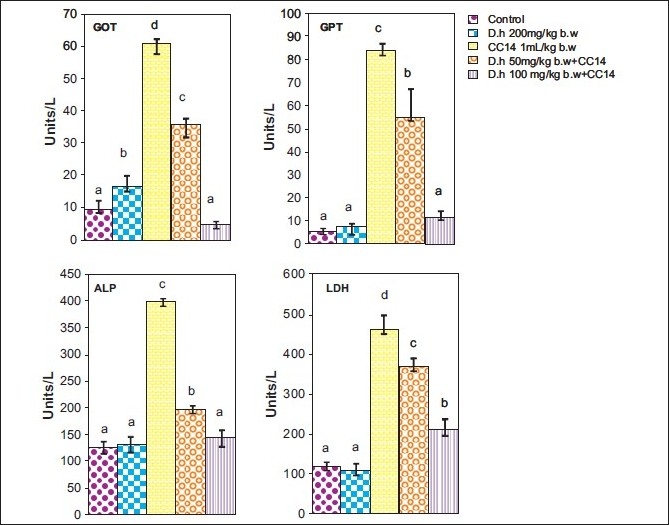
Hepatoprotective action of methanolic extract (multiple dose) of *D. hamiltonii* against CCl_4_ hepatotoxicity: serum enzyme profile

**Figure 3:**
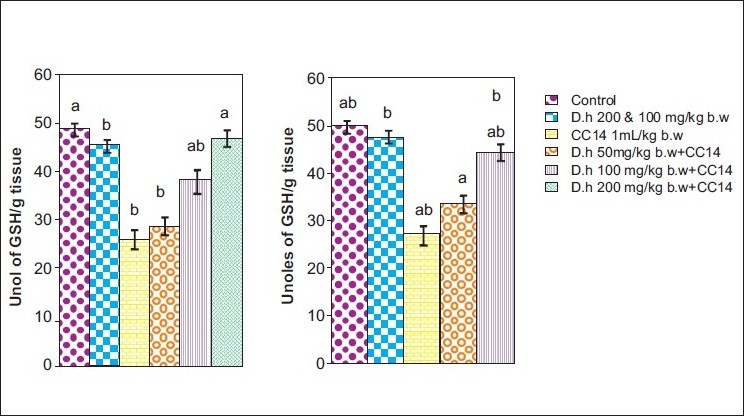
Hepatoprotective action of methanolic extract of *D. hamiltonii* against CCl_4_ hepatotoxicity. GSH profile (a) single dose and (b) multiple dose

**Figure 4:**
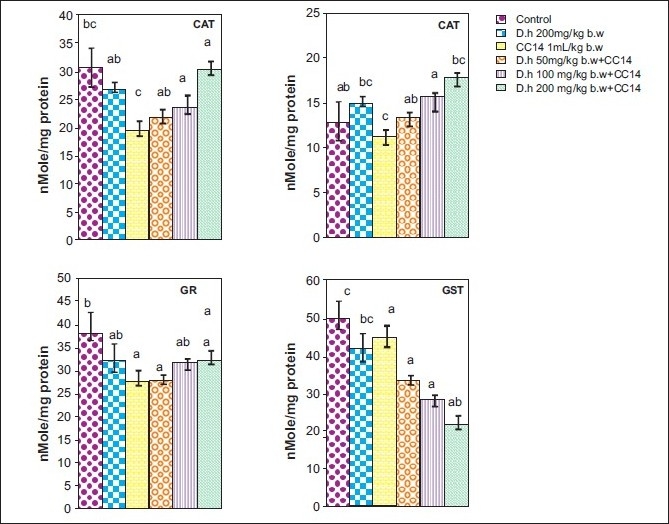
Hepatoprotective action of methanolic extract (single dose) of *D. hamiltonii* against CCl_4_ hepatotoxicity: hepatic antioxidant enzyme profile

**Figure 5:**
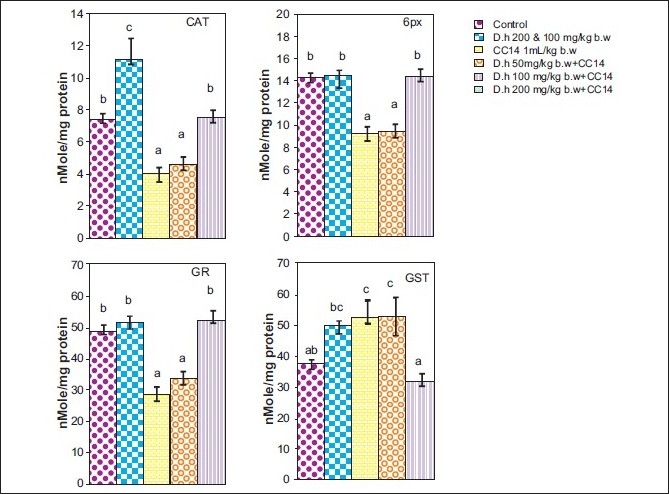
Hepatoprotective action of methanolic extract (multiple dose) of *D. hamiltonii* against CCl_4_ hepatotoxicity: hepatic antioxidant enzyme profile

**Figure 6:**
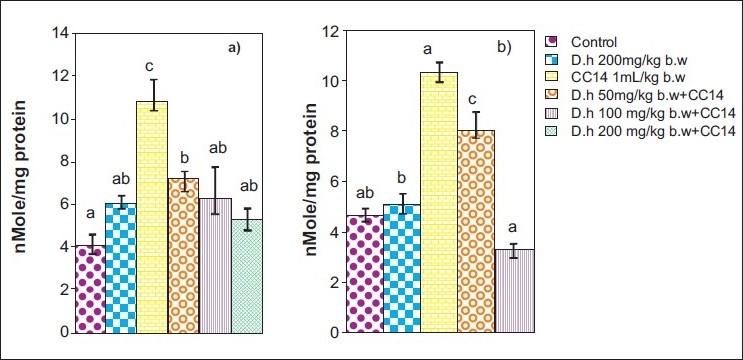
Hepatoprotective action of methanolic extract of *D. hamiltonii* against CCl_4_ hepatotoxicity. LPO profile (a) single dose and (b) multiple dose

Rats treated with CCl_4_ showed significant hepatic damage as observed from increase in serum enzymes (SGOT, SGPT, LDH, ALP) and lipid peroxidation [Figures 1–3] and depletion of glutathione (GSH), catalase, glutathione peroxidase, glutathione reductase, and increase in glutathione transferase [Figures 4–6]. However, pretreatment of rats with Dh extract (single/multiple dose) followed by CCl_4_, afforded protection by lowering the serum enzymes. In addition, pretreatment of extract normalized the level of antioxidant enzymes [Figures 4 and 5]. Protection was observed maximally with the highest dose of the extract [Figures 1 and 2].

### Histopathological studies

**Figure 7:**
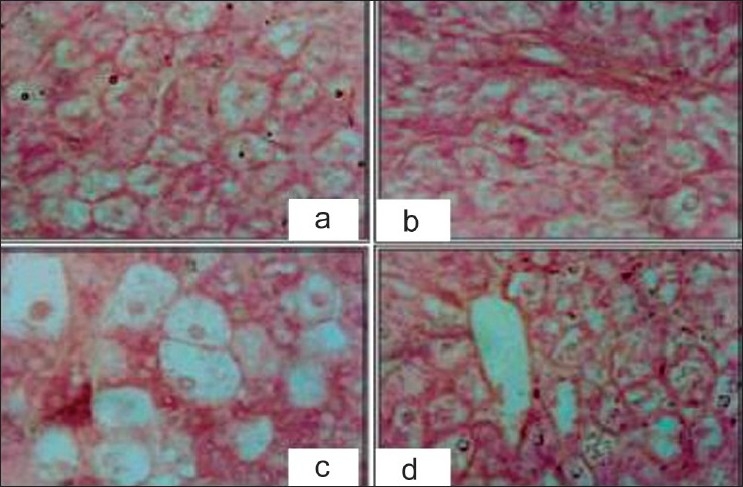
Hepatoprotective action of methanolic extract of *D. hamiltonii* against CCl_4_ hepatotoxicity: liver histopathology

**Figure 8:**
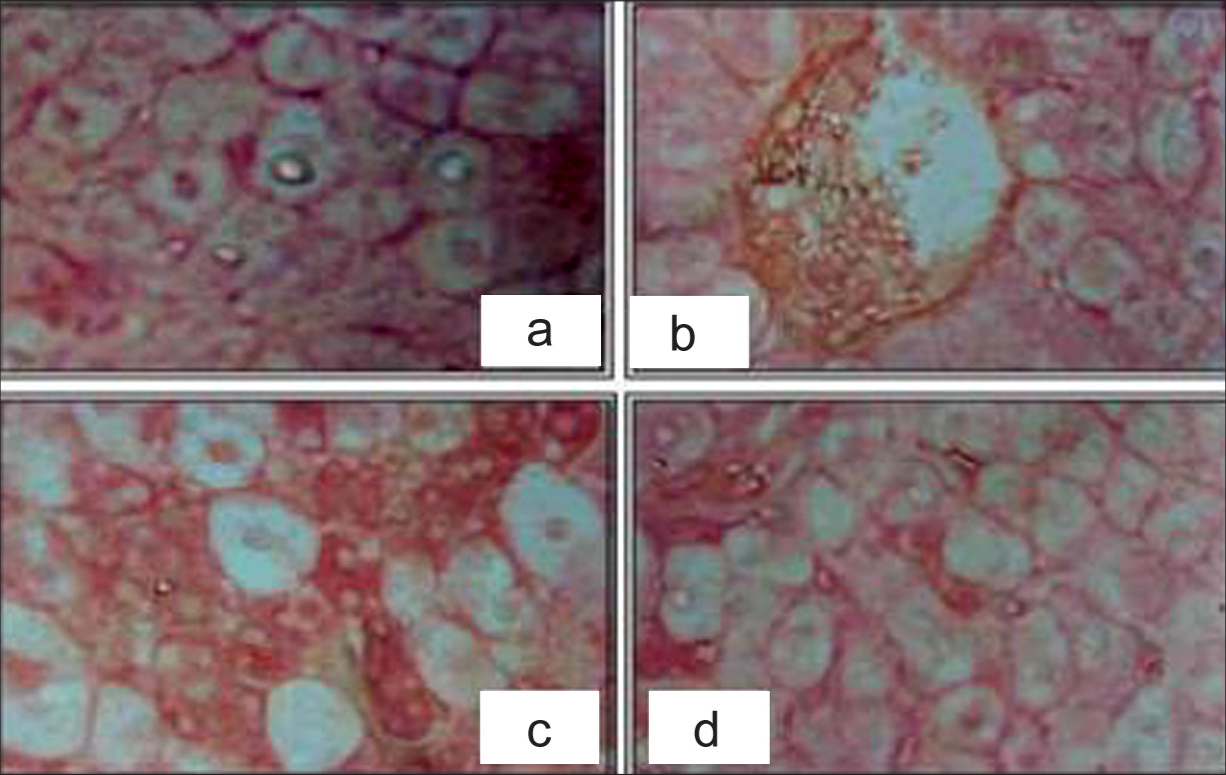
Hepatoprotective action of methanolic extract of *D. hamiltonii* against CCl_4_ hepatotoxicity: liver histopathology

Histopathological studies shows that the liver cells of rats intoxicated with CCl_4_ have high damage, as characterized by the cell vacuolation, pyknotic, degenerated nuclei and wall of bile capillaries compared to the liver of normal animals [Figures 7 and 8]. The shape of the liver is completely damaged; wide spaces are formed at some sinusoids.

In the liver cells of rats treated with single dose of *D. hamiltonii* extract and intoxicated with CCl_4_, the nucleus are not very clear as compared to normal hepatocytes, but when compared to the CCl_4_-damaged ones the number of hepatocytes with normal nucleus are much more [Figure 7]. Endothelium is disrupted at places. Hepatic cells adjoining to intralobular vein show atrophy. Pyknotic nucleus and vaculaton in cytoplasm are observed to be low.

The hepatic cells of rats treated with multiple dose of *D. hamiltonii* extract and intoxicated with CCl_4_ are radially arranged. The vacuolation is present, but is very much similar to that of normal [Figure 8]. The hepatic cells are mostly normal but with few vacuoles and some damaged cells, but no pyknosis in the nucleus could be seen.

## Discussion

In general CCl_4_ has been extensively used as model system to study the hepatic damage and used as an indicator of protective activity of newly discovered drugs.[12] It is well known that CCl_4_ is biotransformed by CytP450 system to produce trichloromethyl-free radical, which further undergoes reduction to form a trichloromethylperoxyl radical, which leads to lipid peroxidation and finally leads to cell death.[12,21] Therefore, leakage of large quantities of enzymes into the blood stream is often associated with massive necrosis of the liver.[22,23] In agreement with results obtained in previous investigation,[11,22] our present study elicited a significant increase in the activities of serum enzymes and decreased the levels of antioxidant enzymes on exposure of rats to CCl_4_ indicating considerable hepatocellular injury. Pretreatment of rats with the Dh root extract (single and multiple dose) reversed the changes produced by CCl_4_. Subsequent recovery toward normalization of serum and antioxidant enzymes strongly suggests the possibility of root extract being able to condition the hepatocytes so as to cause accelerated regeneration of parenchyma cells, thus protecting against membrane fragility and decreasing the leakage of marker enzymes. Another possibility of normalization of liver from oxidative damage could be triterpenes which have been described as anti-inflammatory agents and are known to induce protein synthesis.[24]

GSH is an important endogenous antioxidant system, known to have key functions in protective process. Due to excessive production of CCl_3_ radical, the reduced glutathione becomes oxidized to GSSG; therefore the amount of GSH in CCl_4_ -treated groups is lowered compared to normal group. However, pretreatment of the root extract increased the level of GSH. Lowered catalase activity in CCl_4_ -treated groups could be due to increased superoxide anions as superoxide anions have been shown to inhibit catalase activity.[25] The elevated level of glutathione transferase (GSH-T) activity may increase glutathione (GSH) synthesis in order to counteract CCl_4_ -induced oxidative stress. Decrease in GPx and Gr leads to an increase in hydrogen peroxide, thereby damaging membrane lipids via Fenton reaction. However on pretreatment of Dh root extracts, the activity of Gpx, Gr, and GSH-T normalized and was comparable to normal groups [Figures 4–6]. Results in Figures 1,2,4, and 5 explain that although both the treatments (single and multiple dose) offer hepatoprotection in dose-dependent manner, but multiple doses at lower concentration (50 mg/kg of body weight) was more effective than single dose (200 mg/kg body weight). The probable mechanism by which the root extract exerts its protective action against CCl_4_-induced hepatocellular metabolic alterations could be by the stimulation of hepatic regeneration through an improved synthesis of protein or interference with the microsomal activation of CCl_4_ and/or its accelerated detoxification and excretion. Dh root extract is reported to be rich in antioxidants.[9,26] Methanolic extract includes many antioxidant molecules viz., vanillin anisaldehyde, borneol, saliycylaldehyde, 2-hydroxy-4methoxybenzaldehyde, and decalepin.[8] The above compounds have the potential to minimize the deleterious effects of free radicals including hydroxyl radicals[8] and thereby can be ranked as hepatoprotective agents. However, which of these components are actually responsible for the antihepatotoxic potential remains to be seen in the future course of our experiments.

Histopathological examination clearly reveals that the extract of *D. hamiltonii* works as hepatoprotectant. Simultaneous treatment of the extract with CCl_4_ exhibits less damage to the hepatic cells as compared to the rats treated with CCl_4_ alone. The sections of the liver pretreated with extract followed by CCl_4_ reveals better hepatoprotecive activity. Negligible damage to a few hepatocytes present in the close vicinity of intralobular vein is observed. Endothelium lining is almost smooth except in few places. Hepatocytes show normal appearance; only some cells show higher numbers of vacuoles in the cytoplasm but no pyknosis in the nucleus could be seen. The results of histopathological parameters and biochemical assays support that *D. hamiltonii* root can be considered to be an effective hepatoprotectant.
